# Potential risks in sentinel lymph node biopsy for cervical cancer: a single-institution pilot study

**DOI:** 10.1186/s12957-020-01905-9

**Published:** 2020-06-18

**Authors:** Hua Tu, Ting Wan, Xinke Zhang, Haifeng Gu, Yanling Feng, He Huang, Jihong Liu

**Affiliations:** 1 Department of Gynecologic Oncology, Sun Yat-sen University Cancer Center, East Dongfeng Road 651, Guangzhou, 510060 China; 2 State Key Laboratory of Oncology in South China, Guangzhou, 510060 China; 3 Collaborative Innovation Center for Cancer Medicine, Guangzhou, 510060 China; 4 Department of Pathology, Sun Yat-sen University Cancer Center, Guangzhou, 510060 China

**Keywords:** Cervical cancer, Sentinel lymph node biopsy, Metastasis, Lymphovascular invasion

## Abstract

**Background:**

Sentinel lymph node (SLN) biopsy is an attractive technique that is widely performed in many oncological surgeries. However, the potential risks in SLN biopsy for cervical cancer remains largely unclear.

**Methods:**

Seventy-five patients with histologically confirmed cervical cancer were enrolled between May 2014 and June 2016. SLN biopsies were performed followed by pelvic lymphadenectomies and all resected nodes were labeled according to their anatomic areas. Only bilateral detections of SLNs were considered successful. Patients’ clinicopathologic feature, performance of SLN detection, and distributions of lymph node metastases were analyzed.

**Results:**

Of the 75 enrolled patients, at least one SLN was detected in 69 (92.0%), including 33 in bilateral and 36 in unilateral. SLNs were most detected in the obturator area (52 of 69 patients, 75.4%) and 26 (37.7%) patients presented SLNs in more than one area of hemipelvis. Lymphovascular invasion was found to be the only factor that adversely influenced SLN detection, while the tumor diameter, growth type, histological grade, deep stromal invasion, and neoadjuvant chemotherapy showed no significant impacts. Patients with lymphovascular invasion showed a significantly higher rate to have unsuccessful detection (90.9% versus 41.5%, *P* < 0.001) and lymph node metastasis (40.9% versus 3.8%, *P* < 0.001) compared with those without. Nodal metastases were confirmed in 11 patients, of whom 9 (81.8%) had lymphovascular invasion and 7 (63.6%) had non-SLN metastasis. The most frequently involved SLNs were obturator nodes (9/11, 81.8%). In addition, the parametrial nodes also have a high rate to be positive (4/11, 36.4%), although they were relatively less identified as SLNs. Besides, 3 patients showed metastases in the laterals without SLN detected.

**Conclusions:**

In cervical cancer, lymphovascular invasion is a significant factor for unsuccessful SLN detection. The risk of having undetected metastasis is high when SLN is positive; therefore, further lymphadenectomy may be necessary for these patients.

## Background

In the management of cervical cancer, sentinel lymph node (SLN) biopsy is a promising technique which may become an alternative to conventional pelvic lymphadenectomy. SLN refers to the first node that receives lymphatic drainage from the primary tumor [1]. Theoretically, systematic lymphadenectomy can be omitted if the SLNs are confirmed free of metastasis. This concept has been widely validated in the management of breast cancer and melanoma [2, 3]. In cervical cancer, numerous studies had also proven the high sensitivity of SLN biopsy in predicting nodal metastasis [4–9].

Although the value of SLN biopsy in cervical cancer has been well demonstrated, there is no consensus on what should be done when SLNs are successfully detected. The European Society of Gynecological Oncology/European Society for Radiotherapy and Oncology/European Society of Pathology guidelines recommended performing SLN biopsy before lymphadenectomy and assessing SLNs with a frozen section to immediately triage patients toward radical hysterectomy with lymphadenectomy or concurrent chemoradiotherapy [10]. They did not recommend SLN biopsy alone outside prospective clinical trials. However, the National Comprehensive Cancer Network (NCCN) guidelines did not make the same recommendation [11]. In clinical practice, some gynecologists are still used to perform systematic lymphadenectomy for patients with positive SLN identified on intraoperative assessment [12], whereas others directly exempt lymphadenectomy for all patients and plan chemoradiotherapy for those presenting positive SLN on final pathology. A recent international survey by the Gynecologic Cancer Intergroup clearly reflected these divergences in gynecologists’ attitudes toward SLN biopsy [13]. Evidence remains lacking to resolve these divergences and it is unclear how much risks will be taken if lymphadenectomy was completely replaced by SLN biopsy.

Furthermore, the uterus is a midline organ with bilateral lymphatic drainage, thus a successful detection should harvest at least one SLN in each hemipelvis. However, even with the newly developed tracers and probes [14], unilateral SLN detection remains a common phenomenon with nonnegligible incidence. Unilateral detection will lower the sensitivity of SLN technique and indicate at least a unilateral pelvic lymphadenectomy [7]. A previous study found that the SLN technique showed a lower detection rate and sensitivity in patients with a tumor larger than 2 cm [15]. However, the risk factors of unsuccessful SLN detection remain insufficiently understood.

Here, we present the results of a single-center prospective study on SLN biopsy for cervical cancer. This study was an exploration prior to a randomized controlled trial comparing pelvic lymphadenectomy and SLN biopsy alone in cervical cancer (ClinicalTrials.gov identifier NCT02642471). The aim of this exploratory study was to evaluate the potential risks in SLN biopsy and formulate a proper strategy for the clinical trial.

## Methods

### Patients

Between May 2014 and June 2016, patients with International Federation of Gynecology and Obstetrics (FIGO 2009) stage IA2-IIB cervical cancer treated in Sun Yat-sen University Cancer Center were enrolled with the approval of Institutional Review Board. All patients had a histologically confirmed diagnosis of invasive cervical cancer and signed informed consent for radical surgery involving SLN procedure. Computed tomography (CT) or magnetic resonance imaging (MRI) examinations were performed to assess the retroperitoneal lymphatic status. The exclusion criteria were as follows: intention of fertility-sparing, no residual cervical tissue for tracer injection, suspected metastatic node on CT/MRI examination, and a history of prior subtotal hysterectomy.

### Surgical procedures

At the beginning of surgeries, all patients were injected tracers at 3 and 9 o’clock positions of the cervix. The tracers used in this study included methylene blue (2 ml:20 mg, Jiangsu JUMPCAN Pharmaceutical Co, China) and carbon nanoparticles suspension (1 ml:50 mg, Chongqing LUMMY Pharmaceutical Co, China). The depth of injection was about 0.3 to 0.5 cm and the time length required for injection was at least 2 min. Immediately after injection, all patients underwent surgical procedures including SLN biopsy followed by systematic pelvic lymphadenectomy, and radical hysterectomy with or without bilateral salpingo-oophorectomy. Paraaortic lymphadenectomy was performed only if preoperative histological grade was 3 or common iliac node metastasis was detected. No restriction for surgical approaches was applied.

To investigate the potential rule of SLN distribution, we labeled the pelvic nodes according to their anatomic areas including common iliac, external iliac, internal iliac, parametrial, and obturator. The procedures of SLN biopsy involved exploratory of the iliac vessel areas and the parametrial and obturator spaces. If no stained node were detected in these areas, the pre-sacral and paraaortic areas would also be inspected. All stained nodes were identified as SLNs and dissected. According to the performance of SLN detection, we classified bilateral detection as successful, unilateral, and failed detections together as unsuccessful.

### Pathological procedures

During operation, SLNs were delivered to pathology room for frozen section examination, which were performed simultaneously by two pathologists. SLNs were bisected and one section was taken from the maximum surface of each half node and examined after staining with hematoxylin and eosin. The rest tissue of SLNs was fixed for final pathological examination, in which each paraffin block was sectioned at 2-mm intervals and then submitted for hematoxylin and eosin staining.

Any negative SLN on routine pathological examination would process an ultrastaging protocol, which involved 4 serial sections from each paraffin block, each obtained at 4 levels of 200-μm interval. The first section of each level was stained with hematoxylin and eosin. AE1/AE3 antibody was used for immunohistochemical examination when necessary. All non-SLNs were entirely submitted and blocked following 3-mm intervals and routine hematoxylin and eosin staining. In this study, isolated tumor cells were defined as < 0.2 mm, micrometastasis as between 0.2 and 2 mm, and macrometastasis as > 2 mm.

### Data collection and statistics

The patients’ clinic-pathological characteristics, the information of SLNs, and frozen section examination and final pathology results were recorded. If a diagnosis by frozen section examination was found to be a false-negative, the initial slices for the frozen section examination would be re-examined. The sensitivity of the frozen section examination was calculated as the ratio of patients having both positive frozen section and sentinel metastasis within all patients having sentinel metastases. The 95% confidence intervals for proportions were estimated with the exact binomial distribution method. The difference in detection rate was investigated using Chi-square or Fisher’s exact test and validated with multivariate logistic regression. All statistical analysis was performed using Statistical Product and Service Solutions software (version 16.0, SPSS Inc., Chicago, IL, USA) with a two-sided *P* value of less than 0.05 considered statistically significant.

## Results

### SLN biopsy and lymphadenectomy performance

A total of 75 consecutive patients were enrolled in this study and all were eligible for analysis. The patients’ characteristics were listed in Table 1. The median age of them was 46 years (range 24 to 61). The FIGO 2009 stages were IA2, IB1, IB2, IIA1, IIA2, and IIB in 4, 44, 9, 10, 3, and 5 patients, respectively. Fifteen patients with stage IIA2, IB2, or IIB disease received 2–3 cycles of neoadjuvant chemotherapy prior to surgeries and achieved partial or complete response. The surgical approaches were laparoscopic in 19 and laparotomic in 56 patients. Methylene blue was used alone in 49 patients and carbon nanoparticles suspension in 24, while in 2 patients they were used together. Of the 75 patients, at least one SLN was detected in 69. Thus, the overall detection rate was 92.0% (69/75), with an accumulated number of 414 for SLNs totally dissected (median, 5; range, 0~19).

**Table 1:** The clinic-pathological characteristics of 75 patients with cervical cancer

Clinic-pathological characteristics	Number
Patient age	
≤ 45 years	35
> 45 years	40
FIGO stage	
IA2	4
IB1	44
IB2	9
IIA1	10
IIA2	3
IIB	5
Histological grade	
G1	2
G2	37
G3	36
Histological type	
Squamous cell carcinoma	58
Adenocarcinoma	15
Adenosquamous carcinoma	1
Big cell neuroendocrine carcinoma	1
Neoadjuvant chemotherapy	
Yes	15
No	60
Surgical approach	
Laparotomic	60
Laparoscopic	15

Of the 69 patients with at least one SLN detected, 33 (47.8%) were bilateral and 36 (52.2%) were unilateral (17 on the left side and 19 on the right). The median time from injection to detection was 12 (ranged 1 to 30) min. The most stained nodes were obturator nodes (stained in 52 of 69 patients, 75.4%), followed by external iliac (66.7%), common iliac (28.9%), internal iliac (17.4%), and parametrial nodes (11.5%). Besides, one patient had SLN detected in the pre-sacral area. Twenty-six (37.7%) patients had more than one area of SLNs detected in their hemipelvis. All patients underwent bilateral pelvic lymphadenectomy and 15 underwent additional paraaortic lymphadenectomy as well. A total of 2363 lymph nodes were removed with an average of 31.5 per patient (range, 10 ~ 63).

We classified unilateral (*n* = 36) and failed (*n* = 6) SLN detections together as unsuccessful. Then, the association between detection results and clinic-pathological factors was analyzed. As shown in Table 2, the only factor affecting the detection results was lymphovascular invasion. This result was further validated by multivariate logistic regression, in which lymphovascular invasion remained the only independent predictor of unsuccessful SLN detection (adjusted odds ratio = 12.59, 95% CI 2.42–65.39, *P* = 0.003). The bilateral detection rate in patients with lymphovascular invasion was significantly lower compared with that in patients without (9.1% versus 58.5%, *P* < 0.001). In patients with lymphovascular invasion, only 2 out of 22 achieved successful detection. In addition, the differences by other factors, including neoadjuvant chemotherapy history, tumor diameter, histological grade, deep stromal invasion, and growth type, were not significant.

**Table 2:** Clinic-pathological factors for the performance of SLN detection

	Unsuccessful detection (%)	Successful detection (%)	*P* value
Number of patients	42	33	
Neoadjuvant chemotherapy			
Yes	9 (21.4)	6 (18.2)	
No	33 (78.6)	27 (81.8)	0.727
Histological grade			
G1-2	20 (47.6)	19 (57.6)	
G3	22 (52.4)	14 (42.4)	0.392
Deep stromal invasion			
Yes	23 (54.8)	13 (39.4)	
No	19 (45.2)	20 (60.6)	0.186
Lymphovascular invasion			
Yes	20 (47.6)	2 (6.06)	
No	22 (52.4)	31 (94.0)	< 0.001
Tumor diameter			
≥ 2 cm	26 (61.9)	14 (42.4)	
< 2 cm	16 (38.1)	19 (57.6)	0.093
Growth type			
Exophytic	20 (47.6)	22 (66.7)	
Endophytic	14 (33.3)	6 (18.2)	
Ulcerative	8 (19.0)	5 (15.2)	0.229

### Pathological results

A total of 16 metastatic SLNs were finally confirmed in 11 patients, comprising 6 unilateral and 5 bilateral metastases. None of the 6 patients with bilaterally failed SLN detection presented lymph node metastasis. The SLN metastases were detected both by intraoperative and final pathology in 9 patients, while in 2 patients they were detected by final pathology only. According to the standard, the SLN metastases were defined as macrometastases, micrometastases, and isolated tumor cells in 9, 1, and 1 patient, respectively. The 11 patients with nodal metastasis were numbered and their clinico-pathological data are listed in Table 3, showing that these patients were characterized by young age (median: 32 years), high incidence of deep stromal invasion (100%, 11/11), and lymphovascular invasion (81.8%, 9/11). For patients with lymphovascular invasion, the metastatic rate (40.9%, 9/22) was significantly higher than those without (3.8%, 2/53).

**Table 3:** The clinic-pathological feature of the 11 patients with lymph node metastasis

Patient number	Age	FIGO 2009 stage	Tumor volume (cm^3^)	Histological grade	Histological type	Deep stromal invasion	Lymphovascular invasion	Metastasis type
1	31	IB2	3 × 4 × 4.5	2	Adenocarcinoma	Yes	Yes	Macrometastasis
2	30	IB1	3 × 2.5 × 1	2	Adenocarcinoma	Yes	Yes	Macrometastasis
3	32	IA2	2.5 × 2 × 1	2	Squamous carcinoma	Yes	Yes	Micrometastasis
4	32	IB2	2.5 × 2 × 1	2	Adenocarcinoma	Yes	No	Macrometastasis
5	51	IB1	1.5 × 1.5 × 1	2	Squamous carcinoma	Yes	Yes	Macrometastasis
6	32	IB1	2.5 × 2 × 1.5	2	Squamous carcinoma	Yes	Yes	Macrometastasis
7	31	IB2	3 × 3 × 4.0	2	Squamous carcinoma	Yes	Yes	Macrometastasis
8	44	IB1	3.5 × 3 × 1.3	2	Squamous carcinoma	Yes	Yes	Macrometastasis
9	37	IB1	1.8 × 0.9 × 0.9	3	Squamous carcinoma	Yes	Yes	Isolated tumor cells
10	55	IB1	2.5 × 2 × 1.3	3	Squamous carcinoma	Yes	No	Macrometastasis
11	58	IB1	2 × 2 × 1	3	Squamous carcinoma	Yes	Yes	Macrometastasis

The details on SLN detection and pathological examination of the 11 patients are listed in Table 4. Additional non-SLN metastases were observed in 7 patients (No. 1, 3, 4, 6, 7, 8, and 11), which were only revealed by pelvic lymphadenectomy. Accordingly, the risk of having residual metastases after a positive SLN biopsy was 63.6%. No patient with negative SLN presented ipsilateral nodal metastasis, while three patients presented metastases on the hemipelvis without SLN detected. The most frequently involved nodes were obturator (9/11, 81.8%) and external iliac nodes (6/11, 54.5%), which was similar to the tendency of SLN distribution. Notedly, the parametrial nodes also had a high rate to be involved (4/11, 36.4%), although they were relatively less identified as SLN. The pre-sacral SLN detected in patient No. 7 was also confirmed to be metastatic. No patient had paraaortic node metastasis and the overall rate of nodal metastasis in the whole cohort was 14.7% (11/75).

**Table 4:** The details of SLN detection and pathological examination of the 11 patients with lymph node metastasis

Patient Number	Number of SLNs		SLN lateral	Metastatic lateral	Locations of SLNs	Metastases on frozen examination	Metastases on final pathology	
	Total	Metastatic					SLNs	Non-SLNs
1	3	3	Bilateral	Bilateral	Left: E; right: O	Left: E; right: O	Left: E; right: O	Right: M
2	12	3	Bilateral	Right	Left: E, O; right: M	Right: M	Right: M	—
3*	8	1	Bilateral	Bilateral	Left: E, C; right: O	None	Left: E	Right: E
4**	5	1	Right	Bilateral	Right: E, O	Right: E	Right: E	Left: O, M
5	15	1	Left	Left	Left: E, O	Left: O	Left: O	—
6**	2	1	Left	Bilateral	Left: O	Left: O	Left: O	Right: O
7	9	2	Bilateral	Bilateral	S; left: O; right: O	S; right: O	S; right: O	Left: O
8	1	1	Left	Left	Left: E	Left: E	Left: E	Left: M
9*	1	1	Right	Right	Right: O	None	Right: O	—
10	3	1	Bilateral	Left	Left: E; right: E	Left: E	Left: E	—
11	0	0	Left	Left	Left: O	Left: O	Left: O	Left: E

*E* external iliac, *O* obturator, *C* common iliac, *M* parametrial, *S* pre-sacral

*Patients with false-negative frozen section examination

**Patients with unilateral SLN detection have bilateral node metastases

With the aid of ultrastaging procedures, two patients (No. 3 and 9) were found to have occult metastases in SLNs which were omitted by frozen section examination. On the re-review of initial slices of the half-SLNs for frozen section examination, as before, neither of them showed metastasis. However, micrometastasis or isolated tumor cells were detected in their other half-SLNs by serial section examination. Therefore, the sensitivity of frozen section examination in our institution was 81.8% (9/11, 95%CI 47.8–97.8%), with 100% (9/9) for macrometastasis and 0% (0/2) for micrometastasis and isolated tumor cells.

### Follow-up and recurrence

After surgeries, all treatments were planned according to patients’ pathological risks. Standard concurrent chemoradiotherapy was scheduled if patients had any of the high risks including parametrial invasion, positive surgical margins, and/or lymph node metastasis. In absence of any high risk, 3 to 4 cycles of chemotherapies were administrated to patients having prior neoadjuvant chemotherapy. Up to May 2020, the median follow-up time was 53 months (range 34–72), calculated by reverse Kaplan-Meier method. Five patients experienced recurrence, including 2 in the lung, 1 in the groin, 1 in the vaginal stump, and 1 in mediastinal lymph nodes. Their FIGO stages at initial diagnosis were IB2 in 2, IIA2 in 1, and IIB in 2 patients. Notedly, 4 of them had bilaterally failed SLN detection and one had positive SLN. The treatments after recurrences included pelvic exenteration, inguinal lymphadenectomy, and palliative chemotherapy. Up to the last follow-up, no retroperitoneal node recurrence was observed. Two patients died from the disease; one had lung metastasis treated by chemotherapy and another had vaginal stump recurrence treated by pelvic exenteration.

### Discussion

This is a pilot work prior to a randomized controlled study (NCT02642471) aiming at the validation of SLN biopsy in cervical cancer, conducted by the Chinese South-East-Middle (CSEM) Cooperative Group of Gynecological Oncology. The diagnostic value of SLN technique has been verified by previous studies, with a meta-analyzed sensitivity of 91.4% in early-stage cervical cancer [16]. However, no consensus has been achieved on the management subsequent to SLN biopsy [13]. In the latest FIGO staging system for cervical cancer, patients with lymphatic metastasis are classified as stage IIIC and should be treated with concurrent chemoradiotherapy [17]. The safety and benefits of omitting lymphadenectomy in SLN-negative patients remain to be determined in prospective trials (SENTIX = NCT02494063, CSEM010 = NCT02642471, SENTICOL III = NCT03386734) [18, 19]. Yet, in many institutions, gynecologists have been accustomed to replacing lymphadenectomy with SLN biopsy and waiting for final pathology only. This policy was based on the hypothesis that chemoradiotherapy alone is equally efficient as lymphadenectomy combined with chemoradiotherapy when nodal metastasis occurs in early-stage patients. However, this hypothesis has not been verified in a randomized controlled trial.

An obvious concern is, if the patients have chemoradiotherapy resistance, the undetected metastatic nodes may survive and become the sources of recurrence. On the other hand, the extent of metastases is usually beyond the level that positive SLNs locate, so it will be difficult to formulate precise radiation coverage without the information from lymphadenectomy. In our study, the risk of having undetected metastasis after positive SLN biopsy was 63.6%, and it was 66.7% if positive SLNs were found on frozen section examination. Therefore, additional lymphadenectomy based on frozen section examination of SLN might be necessary.

In our study, frozen section examination successfully detected all macrometastases; however, it missed all micrometastases and isolated tumor cells. This result accorded with the findings from previous studies. However, there is no consensus on the clinical significance of SLN micrometastases and whether this indicates the necessity of pelvic lymphadenectomy remains unanswered. In a histological study by Barranger et al. [20], non-SLNs were also examined by ultrastaging techniques including serial sectioning and immunohistochemistry; however, none of the 106 non-SLNs was found to be metastatic. A similar finding was confirmed in the study by Okamoto et al., suggesting that the non-SLNs were seldom involved if the SLNs harbor merely micrometastases [21]. In our cohort, all recurrences occurred in those patients with bilaterally failed SLN detection or positive SLN. None of the non-SLN metastases occurred on the same lateral of SLN micrometastasis or isolated tumor cells, and no pelvic nodal recurrence occurred during the follow-up period. Taken together, these evidences imply that the micrometastasis and isolated tumor cells in SLNs may represent the very beginning of lymphatic spread. For these patients, SLN biopsy is not just diagnostic but also therapeutic, thus lymphadenectomy can be omitted if SLNs had been dissected without macrometastasis.

If frozen section examination has enough accuracy to detect SLN macrometastases, a selective lymphadenectomy policy can be established based on SLN biopsy, which may provide an option to reduce the risk of recurrence arising from residual disease. In a prospective study containing 35 patients, the pelvic lymphadenectomy was omitted in patients with FS-negative SLNs and none of these patients experienced pelvic recurrence in a median follow-up period of 49 months [22]. This study well supported our viewpoint and suggested an important role for frozen section examination in SLN biopsy. However, data on this issue is limited and this viewpoint needs further validation.

As an exploratory study, we included a series of patients that were conventionally recognized not as candidates for SLNB, such as patients with tumors larger than 2 cm or neoadjuvant chemotherapy. The bilateral detection rate was relatively low, which should be partly attributed to our single tracer method. However, we found a significant difference in bilateral detection rates between patients with and without lymphovascular invasion, while no difference was revealed in the comparisons by other factors. This finding suggested that the lymphovascular obstruction by tumorous embolus might be the real reason behind the failure of SLN detection. Besides, we found that the lymphatic drainage from the cervix to SLNs was usually multidirectional as 37.7% of patients simultaneously had multiple groups of SLNs detected in their hemipelvis. Accordingly, there exists a possibility that lymphovascular embolus blocked the drainage toward metastatic nodes, whereas the normal nodes were successfully detected. This selective “shielding effect” by lymphovascular invasion may lead to the omission of metastatic nodes and produce false-negative results. This may also explain why the rate of non-SLN metastasis was so high in the cases with positive SLN. Therefore, in cases with extensive lymphovascular invasion, the pathological results should be paid special attention.

Understanding the particular risk associated with lymphovascular invasion in SLN biopsy may help to optimize the treatment for these patients. Although it is difficult to identify them before radical hysterectomy, it is possible to establish a clinic-pathological model to predict lymphovascular invasion and guide the performance of SLN techniques. Furthermore, we recommend performing multipoint injections on normal cervical area to reduce the influence of lymphovascular invasion, and that patients whose cervix is completely occupied by the tumor should not be considered for SLN technique.

## Conclusion

In cervical cancer, lymphovascular invasion is a significant risk factor for unsuccessful detection of SLNs. The risk of having undetected metastasis is high after positive SLN biopsy therefore lymphadenectomy based on frozen section examination may be necessary.

## Data Availability

The raw data of this paper are available upon reasonable request to the corresponding author.

## Abbreviations

SLN: Sentinel lymph node
NCCN: National Comprehensive Cancer Network
FIGO: International Federation of Gynecology and Obstetrics
CT: Computed tomography
MRI: Magnetic resonance imaging
